# 30 years of *Journal of Synchrotron Radiation*and synchrotron science

**DOI:** 10.1107/S1600577524010798

**Published:** 2025-01-01

**Authors:** S. Samar Hasnain

**Affiliations:** a Molecular Biophysics Group, Life Sciences Building, Institute of Systems, Molecular and Integrative Biology, Faculty of Health and Life Sciences University of Liverpool LiverpoolL69 7ZB United Kingdom

**Keywords:** *Journal of Synchrotron Radiation*, synchrotron radiation, structural biology, EXAFS, imaging, spectroscopy, Nobel prizes

## Abstract

*Journal of Synchrotron Radiation* (*JSR*) came into being with the publication of its inaugural issue in October 1994 that contained 15 full articles comprising 100 pages. Thirty years of *JSR* has coincided with several Nobel Prizes that have arisen from the work undertaken on synchrotron radiation sources, with the first of these awarded to Sir John Walker in 1997, just three years after the launch of *JSR*, and celebrated on the front cover of the journal’s July 1999 issue. This article provides an insight into the motivation as well as the journey of establishing this important journal for the IUCr and the synchrotron radiation community which has continued to grow. We also highlight some of the well cited papers for each of the five-year-periods during these 30 years and demonstrate how the journal has become the natural home for all aspects of synchrotron radiation science and technology.

## Abstract 2

An insight is given into the motivation as well as the journey of establishing this important journal for the IUCr and the synchrotron radiation community.

## 1. Birth of *Journal of Synchrotron Radiation* (*JSR*)

**Figure 1:**
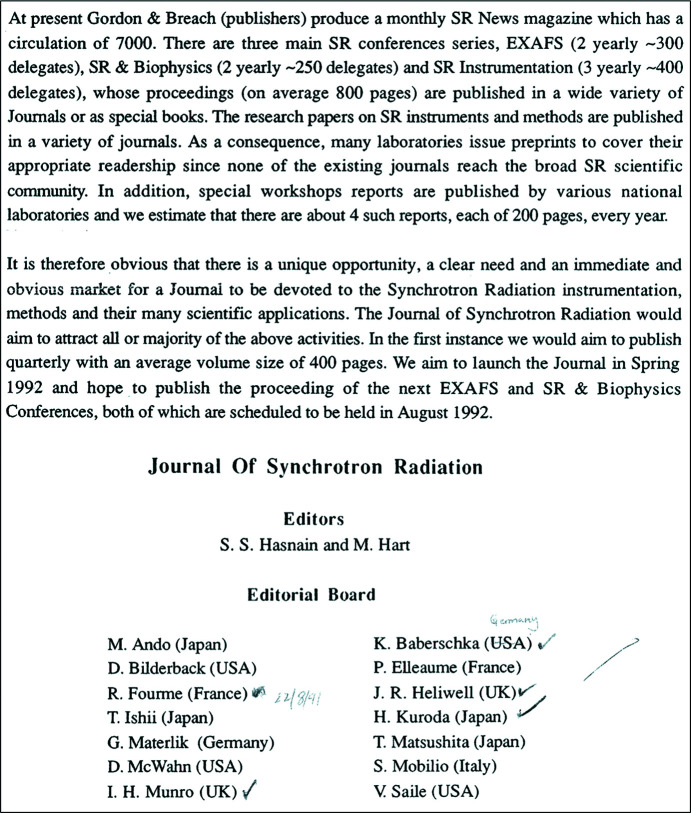
The case for creating the new journal presented to the IUCr’s Finance Committee on 20 August 1991.

**Figure 2:**
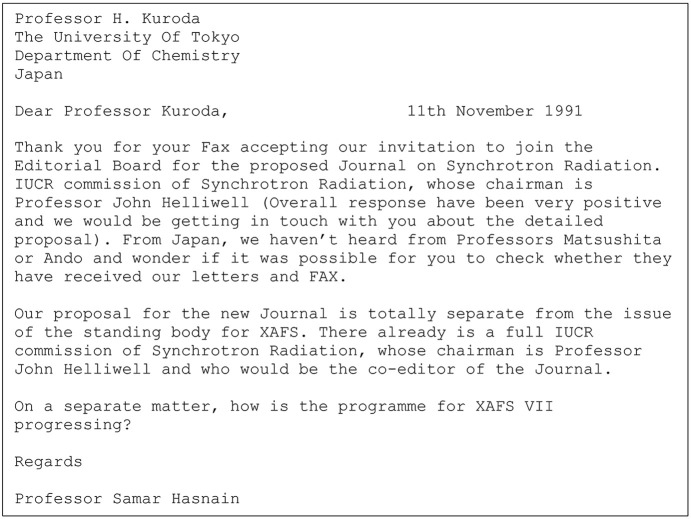
Letter to Professor Kuroda, seeking help.

**Figure 3:**
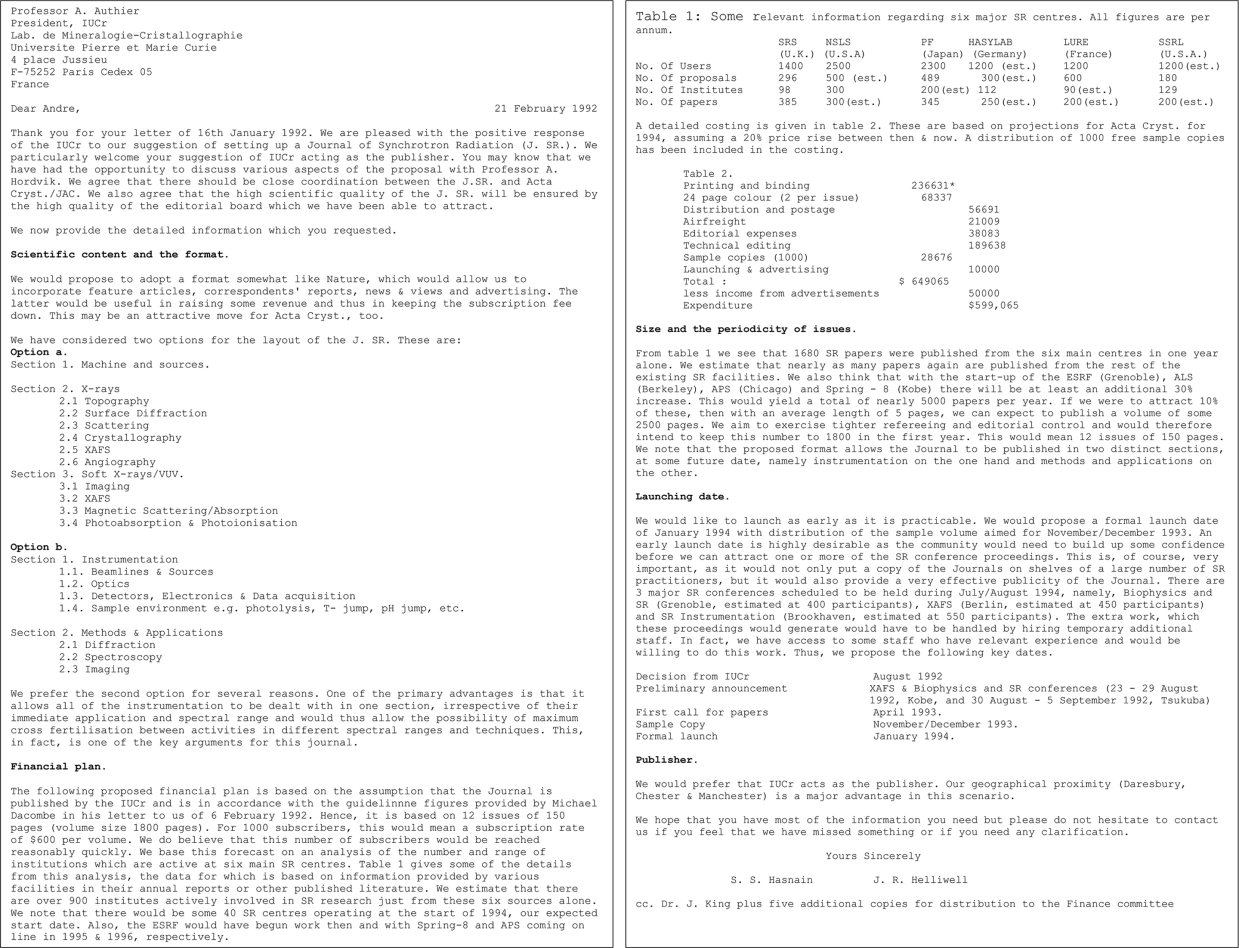
Detailed plan provided to the President of IUCr.

**Figure 4:**
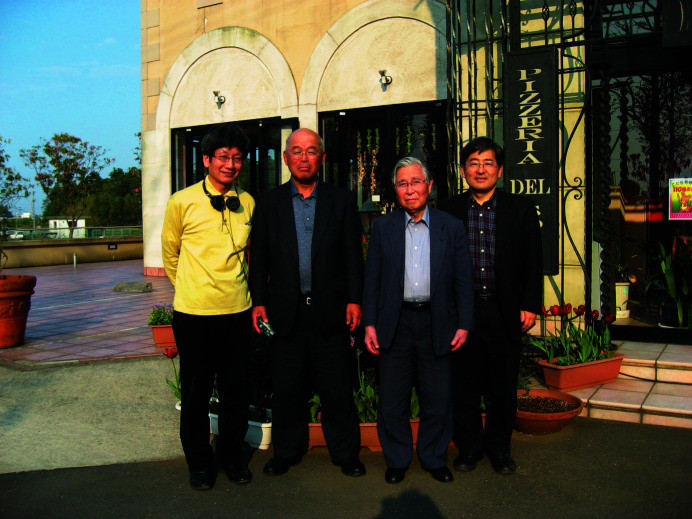
Professor Kohra (second from right) with Yoshiyuki Amemiya (far right) and Masami Ando (far left), who were both founding Co-editors. Professor Amemiya is currently the President of JASRI, responsible for the utilization of SPring-8, SACLA (both located on Harima campus) and NanoTerasu (located in Sendai). He was also a Main Editor of *JSR* from 2015 to 2024.

**Figure 5:**
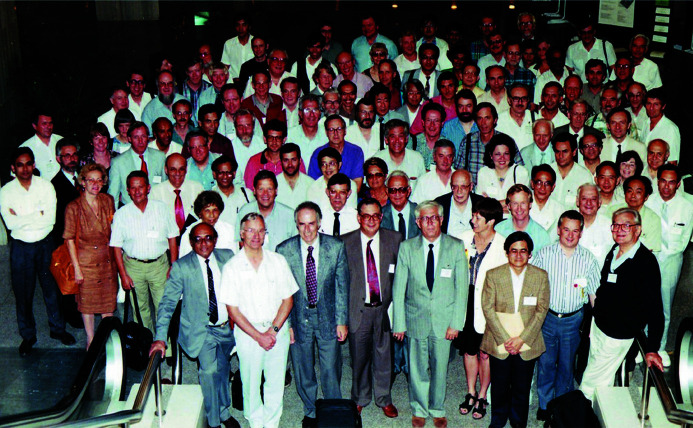
A group photograph of the IUCr General Assembly at Beijing with the Executive Committee members in the front row (from right Hordvick, Hart, Harada, Kalman, Authier, Coppens, Diamond and Chidambaram). Amemiya, Hasnain and Helliwell are in the last row where they are barely visible.

**Figure 6:**
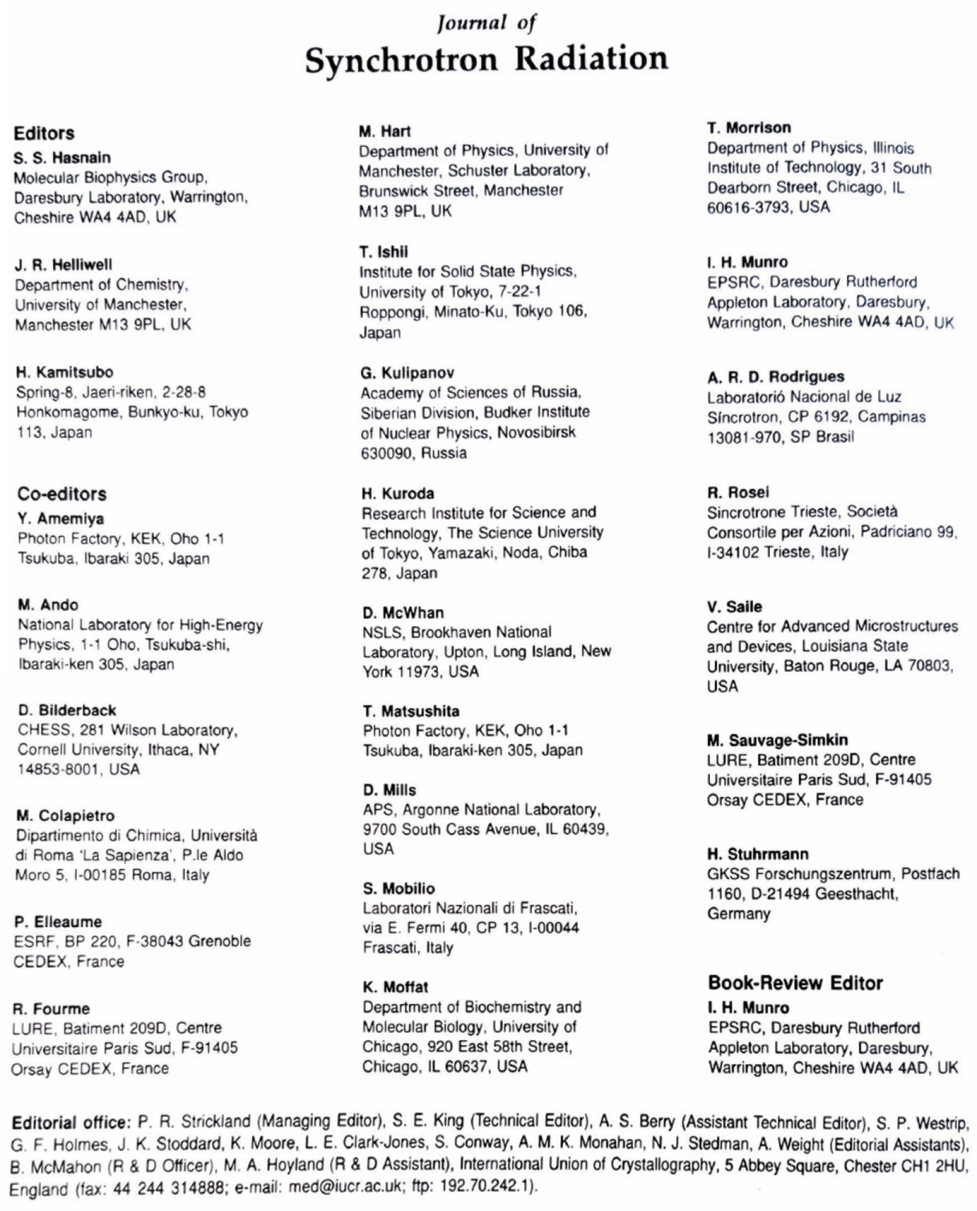
An image of the inside front cover of the inaugural issue of the journal showing the *JSR* founding Editorial Board.

**Figure 7:**
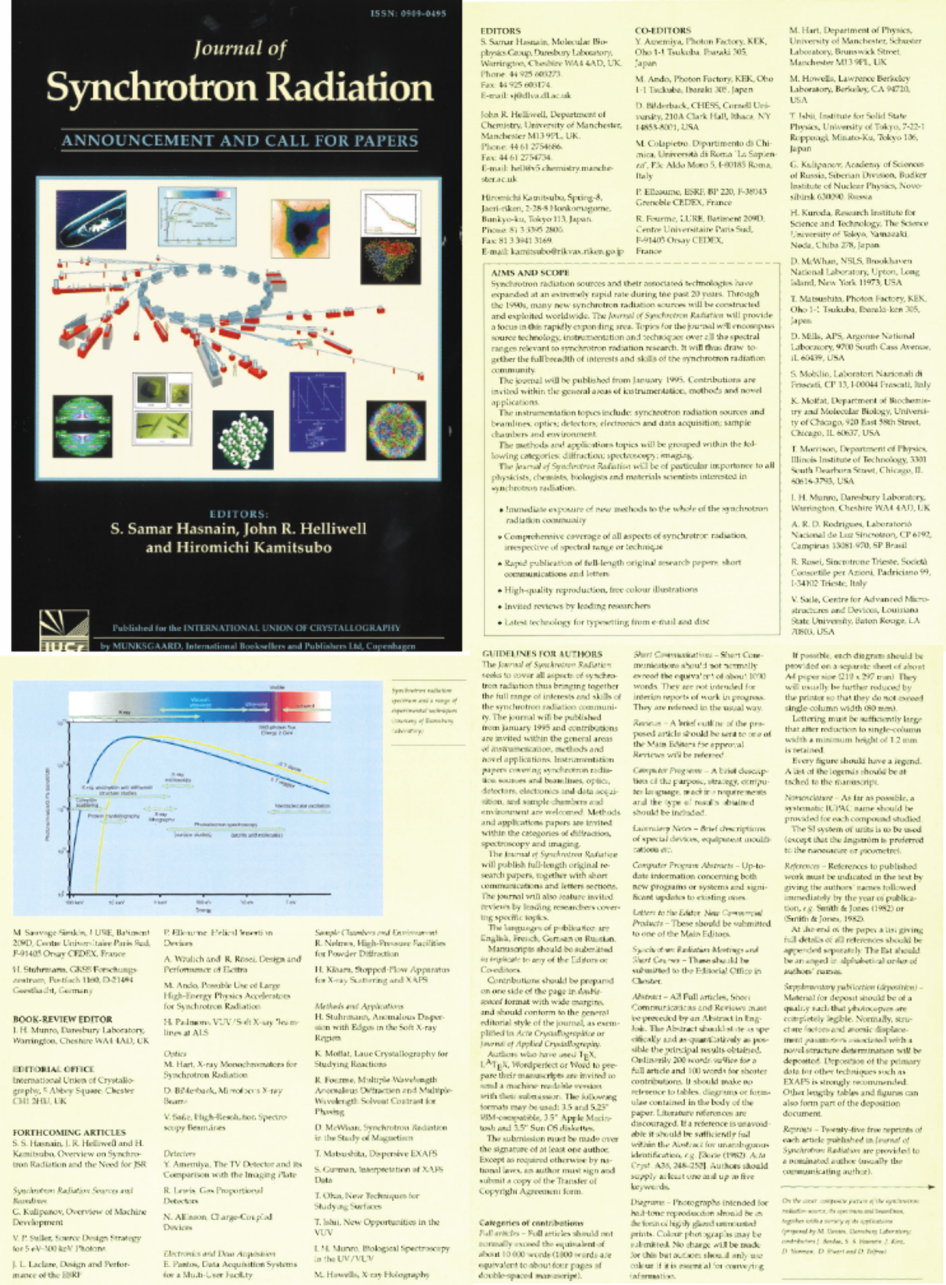
The four-page flyer distributed by the IUCr announcing the launch of the journal to the wider community, with details of the scope, editorial board and forthcoming articles in the early issues.

In 1986–1987 I spent a sabbatical year divided between Birmingham, Alabama (Charlie Bugg’s Laboratory) and Seattle (Lyle Jensen and Ellie Adman). At Seattle I got to know Professor Asbjørn Hordvik from Tromsø University who was a regular visitor to Seattle. My first interaction with the International Union of Crystallography (IUCr) took place in 1987 when, on encouragement from Asbjørn Hordvik and strong support from Michael Woolfson, Chairman of the British Crystallographic Association, I wrote to the IUCr’s Calendar Committee’s Chair Ted Maslen for sponsorship for the *Second International Conference on Synchrotron Radiation and Biophysics (BSR)*. I was pleasantly surprised when I received a letter from Dr Jim King, the first IUCr Executive Secretary (from 1969 to 1993), on 15 April 1988 informing me that IUCr has awarded USD 5000 (which would be over USD 13K today) for the *2nd BSR* conference, to be held in Chester, UK, from 4 to 8 July 1988. This generous sponsorship persuaded me that the synchrotron radiation community would be well served by close alignment with the IUCr. During the next couple of years I explored the idea of a dedicated journal for synchrotron radiation with several colleagues in the UK and abroad. Following initial interactions with IUCr colleagues, I was invited to the IUCr’s head office in Chester on 20 August 1991 to meet the Finance Committee. I presented the case for creating the new journal with a list of potential Co-editors, some of whom had already agreed to serve if such a journal were to be established (Fig. 1 ▸). The meeting was attended by, among others, Charlie Bugg (IUCr Editor-in-Chief at the time), Professor Asbjørn Hordvik from Tromsø University (IUCr Treasurer), Andre Authier (President of the IUCr), Jim King (Executive Secretary of the IUCr) and Mike Dacombe (Technical Editor, IUCr Journals). It emerged during the discussion that it would be good to have John Helliwell as one of the Main Editors, who was the Chairman of the IUCr’s Commission on Synchrotron Radiation which had been established only a year previously in 1990. Below are some key moments in the birth and launch of *JSR*, described in chronological order.

(*a*) On 11 November 1991, I wrote to Professor Haruo Kuroda (University of Tokyo) thanking him for accepting my invitation to join the editorial board and asking for his help with other Co-editors from Japan (Fig. 2 ▸).

(*b*) On 16 January 1992, Andre Authier wrote to us informing us that the IUCr was positive about launching *JSR*. We were asked to provide a detailed proposal in time for the next Finance Committee meeting scheduled to be held in March 1992 in Chester.

(*c*) Before the Finance Committee meeting, John Helliwell and I had an informal meeting with Professor Asbjørn Hordvik at my home. This helped address some of the main anxieties of the committee including potential diversion of papers from *Journal of Applied Crystallography*.

(*d*) On 21 February 1992, we provided a detailed plan to the President of the IUCr (Fig. 3 ▸).

(*e*) We were invited by Jim King to meet the Finance Committee on 21 March 1992 so that our detailed plan could be scrutinized. In addition to the Finance Committee, Professor Mike Glazer was also invited. He was the Editor of *Journal of Applied Crystallography* at the time.

(*f*) Following this, I was invited to make a presentation to the Executive Committee at the ACA meeting in Pittsburgh, USA (9–14 August 1992). I presented a detailed case with the names of a strong team of potential Co-editors. At the evening reception, following my presentation, Professor Alajos Kálmán, who was the Vice President of the IUCr at the time, told my wife that her ‘husband had given a persuasive presentation’ and would get what he had come for. It took a few days before I received the letter on my return to the UK from Professor Andre Authier, President of IUCr, dated 11 August 1992, giving a provisional mandate for *JSR* (Authier, 2009 ▸).

(*g*) It is worth noting that during 1990–1992 we were courted by the *SR News* management (Gordon & Breach) but despite what seemed a slow progress, which was frustrating at the time, we were convinced that the community needed an academic publisher, namely the IUCr. We conveyed this on every possible occasion to the custodians of IUCr, namely the Finance and Executive Committees. Gordon & Breach’s strong interest did indeed catalyse the pace of decision making in 1993.

(*h*) The provisional mandate had come in time for us to hold the first set of meetings of the proposed Editorial Board in Japan during the *XAFS VII* (Kobe, 23–29 August 1992) and *BSR’92* (Tsukuba, 30 August–5 September 1992) conferences, which were organized by Professor Haruo Kuroda and Professor Noriyoshi Sakabe, respectively.

(*i*) Prior to the *XAFS VII* conference, Professor Kuroda, who I had got to know well over the years through our work on the International Advisory Committee for XAFS, had arranged a meeting with Professor Kazutake Kohra, who was the first president of the Japanese Society of Synchrotron Radiation Research (JSSRR), established in 1988. Professor Kohra was the first director of the Photon Factory, which was commissioned in 1982 at Tsukuba, Japan. Professor Kohra listened to my case for establishing *JSR* and there followed some engaging discussion with him and several senior colleagues from Japan (Fig. 4 ▸). JSSRR announced their full support of the journal and that it should be launched with the IUCr. At the conference an international XAFS society was also established and potential association of the community with the IUCr discussed (though this is a separate story for another time).

(*j*) The IUCr’s Executive Committee accepted the proposal unanimously through a postal ballot in May 1993 and took the final decision at the *16th Congress and General Assembly of the International Union of Crystallography* at Beijing, China (21–29 August 1993) (see Fig. 5 ▸). During its final meeting an important intervention was made by Jimpei Harada, an IUCr Executive Member from 1990 to 1996 (Takata, 2023 ▸). He suggested that, in addition to John Helliwell and I, there should be a Main Editor from Japan, and arranged the agreement of Hiromichi Kamitsubo (Ishikawa & Hasnain, 2018 ▸) to be the third Main Editor. Hence the journal was formally launched with its first editorial written by the three of us in October 1994 ( (Hasnain, Helliwell & Kamitsubo, 1994 ▸). We finished the editorial with an invitation to the synchrotron radiation community, ‘The *Journal of Synchrotron Radiation* aims to provide a focus for the whole of the synchrotron radiation community so that the details of any development in one field are easily available to another. Hence, the journal will facilitate the cross-fertilization of ideas and encourage novel applications. In this inaugural issue we have assembled a variety of articles. Many, but obviously not all, aspects of the field of synchrotron radiation and machines, and the associated beamline instrumentation, methods and applications are represented.’ Looking back at the last 30 years, the community has responded, and the journal is flourishing, with the community seamlessly extended to include the free-electron lasers community.

(*k*) Sadly, we were not able to share our happiness and achievements with Dr Jim King who had passed away on 12 April 1993, a month before the Executive Committee had accepted our proposal (Cruickshank & Kurki-Suonio, 1993 ▸). Following Jim’s death, Mike Dacombe and Peter Strickland became the IUCr’s Executive Secretary and Managing Editor, respectively, who ensured the successful launch of the journal with the founding Editorial Board representing 14 synchrotron radiation centres and 8 countries (Fig. 6 ▸). As one would see in the next section, this editorial board had to carry out extra work beyond the regular editorial duties. They had to work hard in soliciting papers, persuading authors that *JSR*, which was not indexed (as for all new journals) in the Science Citation Index, was most suitable for their manuscript, have rigorous refereeing but achieve it with speed so that regular issues could be put together. IUCr produced and distributed a four-page printed flyer to its membership and synchrotron radiation community (see Fig. 7 ▸), highlighting the articles in the initial issues.

## 2. Early years of *JSR*

**Figure 8:**
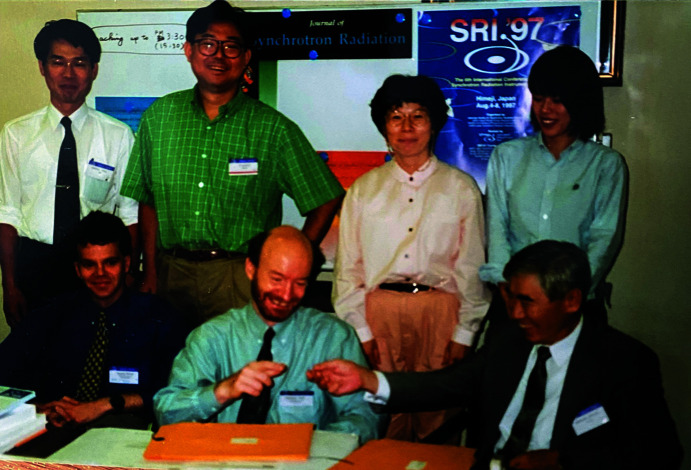
Peter Strickland (bottom, middle) and Hiromichi Kamitsubo (bottom, right) at *SRI’97*.

The early years brought its challenges in persuading authors to submit their manuscripts to *JSR* instead of their regular journals. Although our inaugural issue of October 1994 had 106 pages, the number of pages per issue remained around 60 until the end of 1997. Throughout this time we remained steadfast in providing a helpful but rigorous review process and only accepting papers of high quality. The coverage of the two main synchrotron radiation conferences *SRI’97* (May 1998 issue), *XAFS X* (May 1999 issue) and *XAFS XI* (March 2001 issue) served three important purposes. It attracted authors to the journal in large numbers, bringing a step-change in awareness of the journal to authors and readers rather than a slow growth. It also improved the number of papers and pages of the journal per annum. These proceedings also brought much needed help financially and provided greater exposure of the IUCr’s publishing staff to the wider synchrotron community (see Fig. 8 ▸). In the May 1999 issue, we were able to write: ‘With this issue, we celebrate the fifth anniversary of the journal. Since the launch, approximately 850 papers and 3800 pages have appeared. The journal has published the proceedings of two main synchrotron radiation conferences, *SRI’97* (May 1998 issue) and *XAFS X* (May 1999 issue), where new standards for these proceedings have been set. The journal now features in the top 17% of the Science Citation Index (4800 journals). Its impact factor is greater than that of *Rev. Sci. Instrum.*, *Nucl. Instrum. Methods*, *J. Phys. A* and *J. Phys. C*, and is approaching that of *Phys. Rev. C* and *Phys. Rev. E*. Thus, the *Journal of Synchrotron Radiation* has become clearly established and owes this to the confidence the community has placed in it from its launch.’

**Figure 9:**
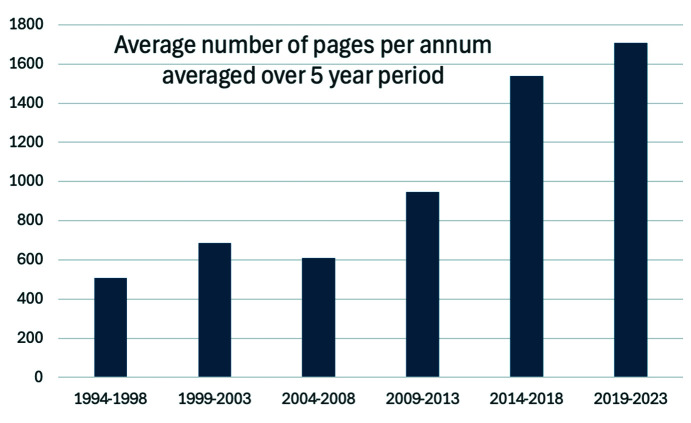
Average number of pages per annum averaged over five-year periods. In 1994 only one issue was published, with 106 pages. In 2024, issues have averaged 280 pages.

To attract a wider audience and authorships, we started specialized thematic issues persuading leaders of the field to contribute reviews, research articles and opinion pieces. An early example of this is the July 1999 issue (Hasnain *et al.*, 1999 ▸) which marked the celebration of the first Nobel Prize associated with synchrotron radiation in 1997 on F1-ATPase (Abrahams *et al.*, 1994 ▸). These measures ensured that we achieved 100 pages per issue. It is pleasing to see that the journal is now regularly attracting more than 200 pages per issue (Fig. 9 ▸).

## 3. Outreach, impact and coverage

**Figure 10:**
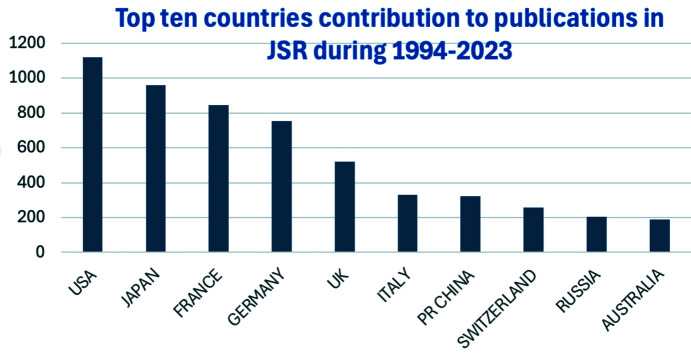
Main countries contributing to articles in *JSR* during 1994–2023.

**Figure 11:**
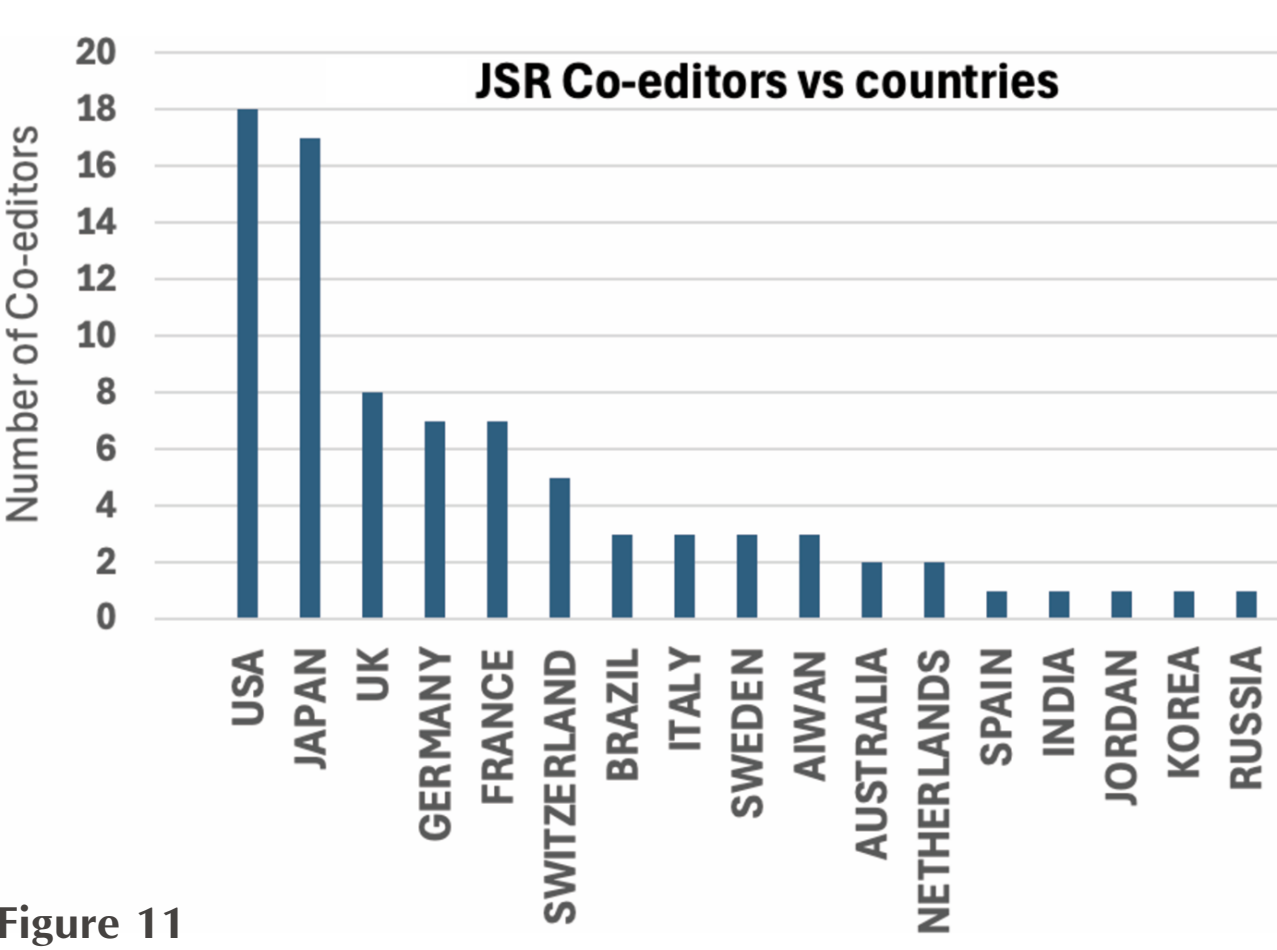
Number of *JSR* Co-editors over the last 30 years.

At the outset, it was important that the journal became the natural home for all aspects of synchrotron radiation science and technology, attracting papers from all the major countries where synchrotron radiation science is taking place. The other important key metric was to ensure that papers of significance were published in the journal. Our hope also was to bring the community together from the machine (accelerator end) to beamlines designer to the end users, leading to constant improvement in the enabling technology. We can see that this has happened, and the journal has certainly contributed to this cohesion and cross-fertilization. Papers have come from around the globe, with USA and Japan contributing the most (Fig. 10 ▸). One can anticipate greater contributions from countries where synchrotron radiation and in some cases XFEL facilities are beginning to thrive, *e.g.* Korea, Taiwan and Brazil now have some of the most advanced sources. The journal has been served by 15 Main Editors so far. They have come from the USA (3), UK (2), Japan (4), EU (5) and India (1). *JSR* has attracted 83 Co-editors from 17 countries reflecting the global reach of the journal (Fig. 11 ▸). Much of the peer review is undertaken by the Co-editors with the help of expert reviewers. The high quality of the published papers reflects the combined efforts of the authors, Co-editors and the reviewers. The supporting information for this article provides details of 151 papers (with a minimum citation of 100) that have attracted a total of 44443 citations, *i.e.* an average citation of 294.

**Figure 12:**
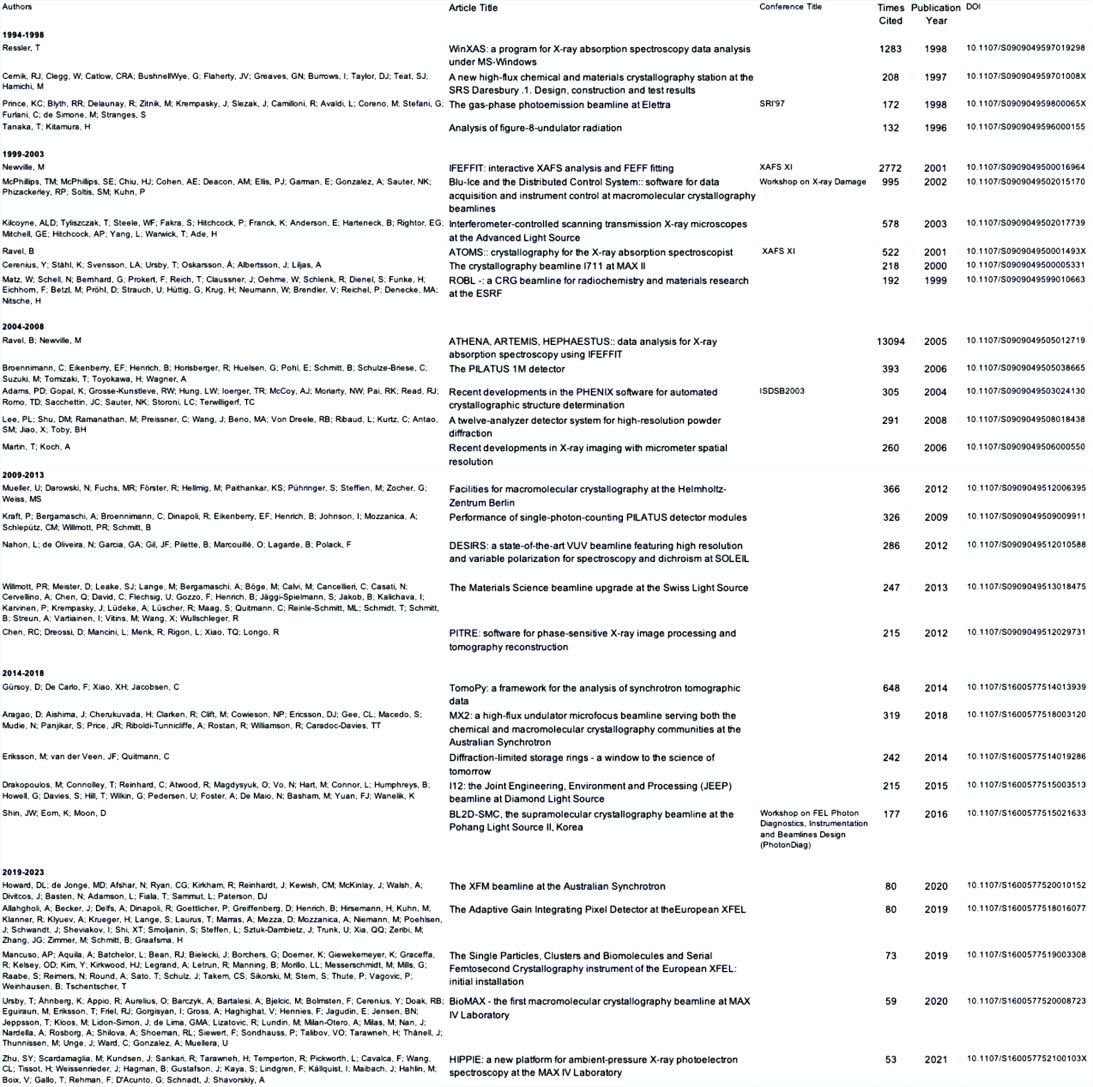
Some of the highly cited papers that have appeared in the journal in each of the five-years windows since the launch of the journal. Several of these are from conference proceedings and specialized workshops.

A closer examination of these papers reveals several important points, some with important implications for the future coverage and attractiveness of the journal. Fig. 12 ▸ provides a selection of highly cited papers in each of the five-years’ time intervals. I have restricted this to five papers per annum (a full list is given in the supporting information). As can be seen, some of these papers are from conference proceedings or issues based on specialized workshops. In the final time window (2019–2023) these papers are dominated by capabilities emerging from diffraction-limited storage rings (DLSRs) [see the September 2014 issue on DLSRs (Eriksson *et al.*, 2014 ▸)] and XFELs. Many of the synchrotron radiation sources are being upgraded to DLSRs including DIAMOND, APS and SPring-8 (Tanaka *et al.*, 2024 ▸); ESRF has already been upgraded, and several XFELs are coming to maturity or are being upgraded. We can expect another burst of activity providing new enabling capabilities to perform diffraction, spectroscopy and imaging experiments at the nano-scale that are currently difficult to contemplate. We hope that authors who would be pioneering these areas either in enabling technology or utilization would continue to support the journal and IUCr. It is inescapable to notice from the supporting information and Fig. 12 ▸ that very few, if any, of these papers report scientific results that are enabled by synchrotron radiation and XFEL sources and instrumentation. This is particularly disappointing given that several Nobel Prizes are now directly associated with the utilization of synchrotron radiation [John Walker in 1997 (Abrahams *et al.*, 1994 ▸; Hasnain *et al.*, 1999 ▸), Rod MacKinnon in 2003 (Hasnain, 2004 ▸; Doyle *et al.*, 1998 ▸; Dutzler *et al.*, 2002 ▸; Dutzler *et al.*, 2003 ▸), Roger Kornberg in 2006 ▸ (Bushnell *et al.*, 2002 ▸; Gnatt *et al.*, 2001 ▸; Hasnain, 2006 ▸), Ramakrishnan, Steitz and Yonath in 2009 (Hasnain, 2007 ▸; Hasnain, 2008 ▸; Hasnain, 2009 ▸; Wimberly *et al.*, 2000 ▸; Selmer *et al.*, 2006 ▸; Ban *et al.*, 2000 ▸; Blaha *et al.*, 2009 ▸; Auerbach-Nevo *et al.*, 2005 ▸; Berisio *et al.*, 2003 ▸), Brian Kobilka in 2012 (Hasnain, 2012 ▸; Rasmussen *et al.*, 2011 ▸; Rasmussen *et al.*, 2011*a* ▸) and Jennifer Doudna in 2020 (Knott *et al.*, 2017 ▸; Knott & Doudna, 2018 ▸)]. We hope that in the coming decade the community at large will consider IUCr journals, including *JSR*, as a suitable home for reporting their exciting scientific results rather than automatically thinking of submitting to *PNAS*, *Nature*, *Science*, *Cell* or their next generation of journals such *PNAS Nexus*, *Nature Communications* or *Science Advances*. It is only through the proactive participation of the next generation of scientists that the IUCr and its journals will flourish. We do not advocate against any of the journals mentioned above but hope that a small fraction, say 10% of the best of science that is enabled by synchrotron radiation and XFEL technology, is reported in *JSR* and other IUCr journals including that which carries IUCr in its name, *IUCrJ*. These could be in the form of current opinion pieces and forward-looking trend articles. A couple of such articles per issue of *JSR* would provide a clear stimulus for attracting high-quality science papers. I urge the community and *JSR* Editors to engage in conversation and find the best way to achieve greater coverage of the science that is enabled by synchrotron radiation and XFEL sources.

## 4. Concluding remarks

*JSR* has been a success story for the synchrotron radiation community and the IUCr. It now is inconceivable to think that synchrotron radiation is not a mainstream activity of the IUCr. This is reflected in several of the Commissions that have been established since the launch of *JSR* which are closely aligned to synchrotron radiation. As mentioned earlier, the Commission on Synchrotron Radiation itself was established in 1990. In 1996 at the 17th Congress and General Assembly several synchrotron radiation related commissions were started including the Commission on XAFS, the Commission on Small-Angle Scattering and the Commission on High Pressure.

The high quality of publication of *JSR* has been maintained over a long period, thanks to authors willing to contribute high-quality papers and reviewers providing rigorous and helpful critique that has generally improved the papers according to feedback from authors. Since 2005, the average impact factor of the journal has been 2.4.

*JSR* also provided greater exposure to the publication staff of the IUCr. As the recently retired Executive Managing Editor, Peter Strickland, puts it, ‘I have been associated with the *Journal of Synchrotron Radiation* since 1993 when I was first introduced to Samar Hasnain and John Helliwell when they visited the IUCr Editorial Offices in Chester, UK. Samar and John worked closely with the editorial staff and the third Founding Editor, Hiromichi Kamitsubo, to launch the journal as planned in 1994. It has been a pleasure to see the journal grow to be highly regarded in the scientific community for the focused and high-quality research that it publishes on synchrotron radiation, XFELs and related techniques. From a personal perspective, working on a journal with global reach has not only given me the chance to visit synchrotron facilities worldwide but it has also given me the chance to meet and interact with many researchers in the synchrotron community. I feel privileged to have been involved with the leading journal in the field of synchrotron science and technology.’ Tony Weight, who is the current Managing Editor of *JSR* and was hired at the time of the launch as an Editorial Assistant to handle the extra work, noted the following, ‘At the beginning, manuscripts were submitted by post in triplicate, and the process of marking up, typesetting and proofing took much longer than today. Over the years, the journal has transitioned fully online, greatly speeding up the workflow. *JSR* has been supported by some of the major synchrotron facilities over the years. APS, DESY, ESRF, SPring-8, Photon Factory, PSI and MAX IV have all helped *JSR*, using its Facility Information pages to share key updates. A notable addition was the introduction of ‘beamline papers’ in 2011, giving an opportunity to synchrotron beamline staff and enhancing the journal’s content. Dealing with the day-to-day aspects of *JSR* has meant working closely with authors and the Editorial Board of *JSR* on a daily basis, and it has been good to get to know them, either by email or in person at conferences or workshops.’

**Figure 13:**
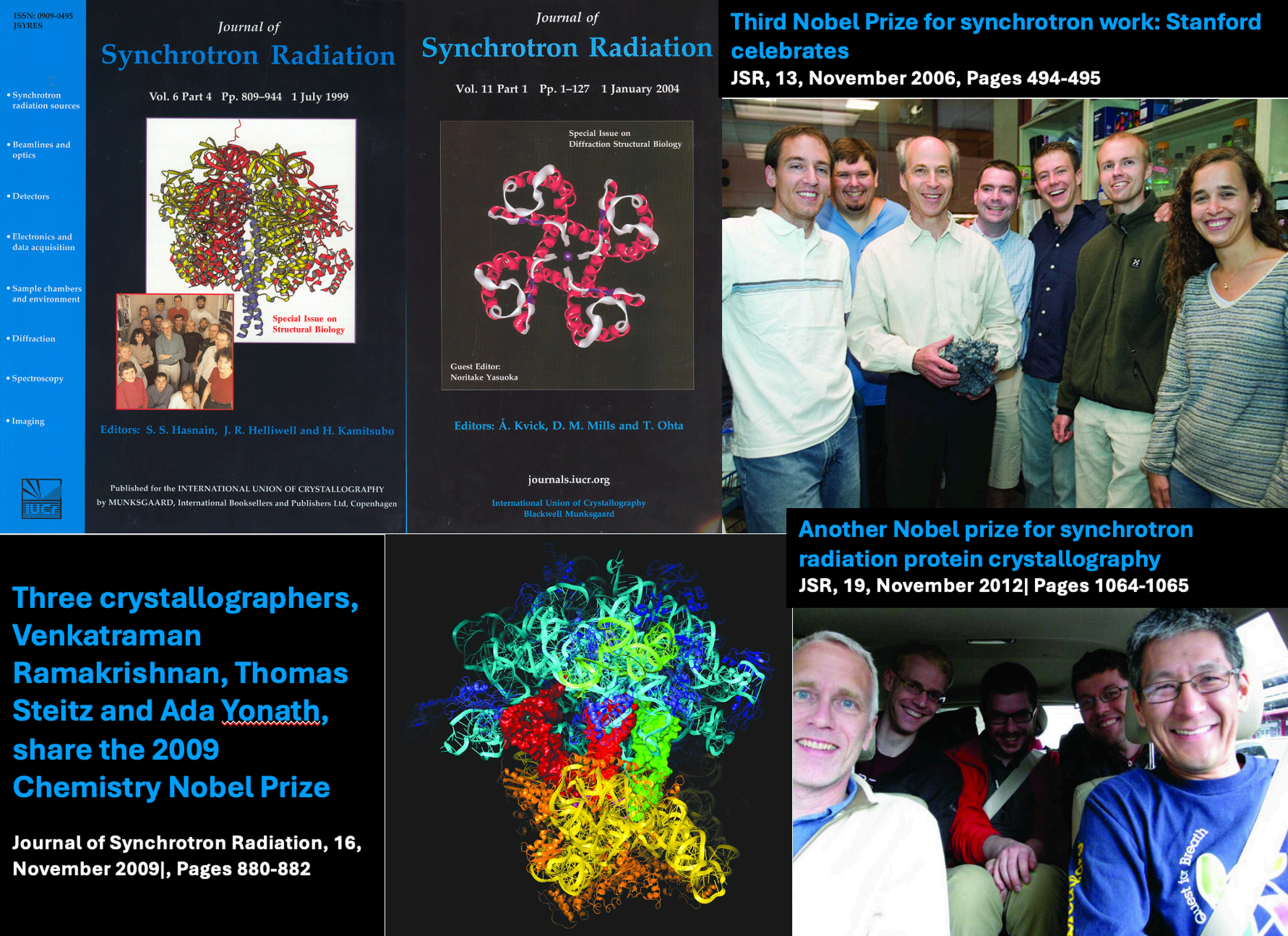
*JSR* has celebrated several Nobel Prizes that have been linked to synchrotron radiation.

The journal has celebrated the success of Nobel Prize winners whose work was linked to synchrotrons either through the front covers, editorials or the current events section. We celebrate this here as Fig. 13 ▸. This image is a reminder to the community that the journal and the IUCr would benefit tremendously if some of the good high-quality science papers were submitted to the IUCr journals including *JSR*. We look forward to the next decade with anticipation of greater success for the journal and the community it represents.

## Supplementary Material

Details of 151 papers (with a minimum citation of 100) that have attracted a total of 44443 citations, i.e. an average citation of 294. DOI: 10.1107/S1600577524010798/me6304sup1.pdf
