# Supplementary material for: 30 years of Journal of Synchrotron Radiationand synchrotron science
Source: J Synchrotron Radiat. 2025 Jan 1;32(Pt 1):1–9. doi: 10.1107/S1600577524010798 (PMC11708846; doi:10.1107/S1600577524010798)
Supplement: Supplementary file 1 [file s-32-00001-sup1.pdf]

JOURNAL OF SYNCHROTRON RADIATION

Volume 32 (2025)

Supporting information for article:

30 years of *Journal of Synchrotron Radiation* and synchrotron science

S. Samar Hasnain

S1. Details of 151 papers (with a minimum citation of 100) that have attracted a total of 44443 citations, i.e. an average citation of 294.

Authors	Article Title	Times Cited	Publication Year	Start Page	End Page	DOI
Ravel, B; Newville, M	ATHENA, ARTEMIS, HEPHAESTUS:: data analysis for X-ray absorption spectroscopy using IFEFFIT	13094	2005	537	541	10.1107/S0909049505012719
Newville, M	IFEFFIT: interactive XAFS analysis and FEFF fitting	2772	2001	322	324	10.1107/S0909049500016964
Ressler, T	WinXAS: a program for X-ray absorption spectroscopy data analysis under MS-Windows	1283	1998	118	122	10.1107/S0909049597019298
McPhillips, TM; McPhillips, SE; Chiu, HJ; Cohen, AE; Deacon, AM; Ellis, PJ; Garman, E; Gonzalez, A; Sauter, NK; Phizackerley, RP; Soltis, SM; Kuhn, P	Blu-Ice and the Distributed Control System:: software for data acquisition and instrument control at macromolecular crystallography beamlines	995	2002	401	406	10.1107/S0909049502015170
Gürsoy, D; De Carlo, F; Xiao, XH; Jacobsen, C	TomoPy: a framework for the analysis of synchrotron tomographic data	648	2014	1188	1193	10.1107/S1600577514013939
Kilcoyne, ALD; Tyliszczak, T; Steele, WF; Fakra, S; Hitchcock, P; Franck, K; Anderson, E; Harteneck, B; Rightor, EG; Mitchell, GE; Hitchcock, AP; Yang, L; Warwick, T; Ade, H	Interferometer-controlled scanning transmission X-ray microscopes at the Advanced Light Source	578	2003	125	136	10.1107/S0909049502017739
Newville, M	EXAFS analysis using FEFF and FEFFIT	561	2001	96	100	10.1107/S0909049500016290
Ravel, B	ATOMS:: crystallography for the X-ray absorption spectroscopist	522	2001	314	316	10.1107/S090904950001493X
Broennimann, C; Eikenberry, EF; Henrich, B; Horisberger, R; Huelsen, G; Pohl, E; Schmitt, B; Schulze-Briese, C; Suzuki, M; Tomizaki, T; Toyokawa, H; Wagner, A	The PILATUS 1M detector	393	2006	120	130	10.1107/S0909049505038665
Mueller, U; Darowski, N; Fuchs, MR; Förster, R; Hellmig, M; Paithankar, KS; Pühringer, S; Steffien, M; Zocher, G; Weiss, MS	Facilities for macromolecular crystallography at the Helmholtz-Zentrum Berlin	366	2012	442	449	10.1107/S0909049512006395
Lengeler, B; Schroer, C; Tümmler, J; Benner, B; Richwin, M; Snigirev, A; Snigireva, I; Drakopoulos, M	Imaging by parabolic refractive lenses in the hard X-ray range	365	1999	1153	1167	10.1107/S0909049599009747
Terwilliger, T	SOLVE and RESOLVE: automated structure solution, density modification, and model building	360	2004	49	52	10.1107/S0909049503023938
Basham, M; Filik, J; Wharmby, MT; Chang, PCY; El Kassaby, B; Gerring, M; Aishima, J; Levik, K; Pulford, BCA; Sikharulidze, I; Sneddon, D; Webber, M; Dhesi, SS; Maccherozzi, F; Svensson, O; Brockhauser, S; Náray, G; Ashton, AW	Data Analysis WorkbeNch (DAWN)	343	2015	853	858	10.1107/S1600577515002283
Tanaka, T; Kitamura, H	SPECTRA: a synchrotron radiation calculation code	341	2001	1221	1228	10.1107/S090904950101425X
Pernot, P; Round, A; Barrett, R; Antolinos, AD; Gobbo, A; Gordon, E; Huet, J; Kieffer, J; Lentini, M; Mattenet, M; Morawe, C; Mueller-Dieckmann, C; Ohlsson, S; Schmid, W; Surr, J; Theveneau, P; Zerrad, L; McSweeney, S	Upgraded ESRF BM29 beamline for SAXS on macromolecules in solution	333	2013	660	664	10.1107/S0909049513010431
Cowieson, NP; Aragao, D; Clift, M; Ericsson, DJ; Gee, C; Harrop, SJ; Mudie, N; Panjikar, S; Price, JR; Riboldi-Tunnicliffe, A; Williamson, R; Caradoc-Davies, T	MX1: a bending-magnet crystallography beamline serving both chemical and macromolecular crystallography communities at the Australian Synchrotron	327	2015	187	190	10.1107/S1600577514021717
Marone, F; Stampanoni, M	Regridding reconstruction algorithm for real-time tomographic imaging	326	2012	1029	1037	10.1107/S0909049512032864
Kraft, P; Bergamaschi, A; Broennimann, C; Dinapoli, R; Eikenberry, EF; Henrich, B; Johnson, I; Mozzanica, A; Schlepütz, CM; Willmott, PR; Schmitt, B	Performance of single-photon-counting PILATUS detector modules	326	2009	368	375	10.1107/S0909049509009911
Aragao, D; Aishima, J; Cherukuvada, H; Clarken, R; Clift, M; Cowieson, NP; Ericsson, DJ; Gee, CL; Macedo, S; Mudie, N; Panjikar, S; Price, JR; Riboldi-Tunnicliffe, A; Rostan, R; Williamson, R; Caradoc-Davies, TT	MX2: a high-flux undulator microfocus beamline serving both the chemical and macromolecular crystallography communities at the Australian Synchrotron	319	2018	885	891	10.1107/S1600577518003120
Adams, PD; Gopal, K; Grosse-Kunstleve, RW; Hung, LW; Ioerger, TR; McCoy, AJ; Moriarty, NW; Pai, RK; Read, RJ; Romo, TD; Sacchettin, JC; Sauter, NK; Storoni, LC; Terwilligerf, TC	Recent developments in the PHENIX software for automated crystallographic structure determination	305	2004	53	55	10.1107/S0909049503024130
Lee, PL; Shu, DM; Ramanathan, M; Preissner, C; Wang, J; Beno, MA; Von Dreele, RB; Ribaud, L; Kurtz, C; Antao, SM; Jiao, X; Toby, BH	A twelve-analyzer detector system for high-resolution powder diffraction	291	2008	427	432	10.1107/S0909049508018438
Pascarelli, S; Boscherini, F; D'Acaptito, F; Hrdy, J; Meneghini, C; Mobilio, S	X-ray optics of a dynamical sagittal-focusing monochromator on the GILDA beamline at the ESRF	291	1996	147	155	10.1107/S0909049596004992
Dyadkin, V; Pattison, P; Dmitriev, V; Chernyshov, D	A new multipurpose diffractometer PILATUS@SNBL	289	2016	825	829	10.1107/S1600577516002411
Strocov, VN; Schmitt, T; Flechsig, U; Schmidt, T; Imhof, A; Chen, Q; Raabe, J; Betemps, R; Zimoch, D; Krempasky, J; Wang, X; Grioni, M; Piazzalunga, A; Patthey, L	High-resolution soft X-ray beamline ADRESS at the Swiss Light Source for resonant inelastic X-ray scattering and angle-resolved photoelectron spectroscopies	287	2010	631	643	10.1107/S0909049510019862
Nahon, L; de Oliveira, N; Garcia, GA; Gil, JF; Pilette, B; Marcouillé, O; Lagarde, B; Polack, F	DESIRS: a state-of-the-art VUV beamline featuring high resolution and variable polarization for spectroscopy and dichroism at SOLEIL	286	2012	508	520	10.1107/S0909049512010588
Buffet, A; Rothkirch, A; Döhrmann, R; Körstgens, V; Kashem, MMA; Perlich, J; Herzog, G; Schwartzkopf, M; Gehrke, R; Müller-Buschbaum, P; Roth, SV	P03, the microfocus and nanofocus X-ray scattering (MiNaXS) beamline of the PETRA III storage ring: the microfocus endstation	273	2012	647	653	10.1107/S0909049512016895
Chubar, O; Elleaume, P; Chavanne, J	A three-dimensional magnetostatics computer code for insertion devices	261	1998	481	484	10.1107/S0909049597013502
Martin, T; Koch, A	Recent developments in X-ray imaging with micrometer spatial resolution	260	2006	180	194	10.1107/S0909049506000550
Amenitsch, H; Rappolt, M; Kriechbaum, M; Mio, H; Laggner, P; Bernstorff, S	First performance assessment of the small-angle X-ray scattering beamline at ELETTRA	257	1998	506	508	10.1107/S090904959800137X
Willmott, PR; Meister, D; Leake, SJ; Lange, M; Bergamaschi, A; Böge, M; Calvi, M; Cancellieri, C; Casati, N; Cervellino, A; Chen, Q; David, C; Flechsig, U; Gozzo, F; Henrich, B; Jäggi-Spielmann, S; Jakob, B; Kalichava, I; Karvinen, P; Krempasky, J; Lüdeke, A; Lüscher, R; Maag, S; Quitmann, C; Reinle-Schmitt, ML; Schmidt, T; Schmitt, B; Streun, A; Vartiainen, I; Vitins, M; Wang, X; Wullschleger, R	The Materials Science beamline upgrade at the Swiss Light Source	247	2013	667	682	10.1107/S0909049513018475
Eriksson, M; van der Veen, JF; Quitmann, C	Diffraction-limited storage rings - a window to the science of tomorrow	242	2014	837	842	10.1107/S1600577514019286
Marcus, MA; MacDowell, AA; Celestre, R; Manceau, A; Miller, T; Padmore, HA; Sublett, RE	Beamline 10.3.2 at ALS: a hard X-ray microprobe for environmental and materials sciences	239	2004	239	247	10.1107/S0909049504005837
Nikitenko, S; Beale, AM; van der Eerden, AMJ; Jacques, SDM; Leynaud, O; O'Brien, MG; Detollenaere, D; Kaptein, R; Weckhuysen, BM; Bras, W	Implementation of a combined SAXS/WAXS/QEXAFS set-up for time-resolved in situ experiments	235	2008	632	640	10.1107/S0909049508023327
Tamura, N; MacDowell, AA; Spolenak, R; Valek, BC; Bravman, JC; Brown, WL; Celestre, RS; Padmore, HA; Batterman, BW; Patel, JR	Scanning X-ray microdiffraction with submicrometer white beam for strain/stress and orientation mapping in thin films	227	2003	137	143	10.1107/S0909049502021362
Bergamaschi, A; Cervellino, A; Dinapoli, R; Gozzo, F; Henrich, B; Johnson, I; Kraft, P; Mozzanica, A; Schmitt, B; Shi, XT	The MYTHEN detector for X-ray powder diffraction experiments at the Swiss Light Source	224	2010	653	668	10.1107/S0909049510026051
Cerenius, Y; Ståhl, K; Svensson, LA; Ursby, T; Oskarsson, Å; Albertsson, J; Liljas, A	The crystallography beamline I711 at MAX II	218	2000	203	208	10.1107/S0909049500005331
Drakopoulos, M; Connolley, T; Reinhard, C; Atwood, R; Magdysyuk, O; Vo, N; Hart, M; Connor, L; Humphreys, B; Howell, G; Davies, S; Hill, T; Wilkin, G; Pedersen, U; Foster, A; De Maio, N; Basham, M; Yuan, FJ; Wanelik, K	I12: the Joint Engineering, Environment and Processing (JEEP) beamline at Diamond Light Source	215	2015	828	838	10.1107/S1600577515003513
Chen, RC; Dreossi, D; Mancini, L; Menk, R; Rigon, L; Xiao, TQ; Longo, R	PITRE: software for phase-sensitive X-ray image processing and tomography reconstruction	215	2012	836	845	10.1107/S0909049512029731
Weitkamp, T; Haas, D; Wegrzynek, D; Rack, A	ANKAphase: software for single-distance phase retrieval from inline X-ray phase-contrast radiographs	212	2011	617	629	10.1107/S0909049511002895
Holton, JM	A beginner's guide to radiation damage	212	2009	133	142	10.1107/S0909049509004361
Cernik, RJ; Clegg, W; Catlow, CRA; BushnellWye, G; Flaherty, JV; Greaves, GN; Burrows, I; Taylor, DJ; Teat, SJ; Hamichi, M	A new high-flux chemical and materials crystallography station at the SRS Daresbury .1. Design, construction and test results	208	1997	279	286	10.1107/S090904959701008X
Meirer, F; Cabana, J; Liu, YJ; Mehta, A; Andrews, JC; Pianetta, P	Three-dimensional imaging of chemical phase transformations at the nanoscale with full-field transmission X-ray microscopy	203	2011	773	781	10.1107/S0909049511019364
Dippel, AC; Liermann, HP; Delitz, JT; Walter, P; Schulte-Schrepping, H; Seeck, OH; Franz, H	Beamline P02.1 at PETRA III for high-resolution and high-energy powder diffraction	201	2015	675	687	10.1107/S1600577515002222
Liu, YJ; Meirer, F; Williams, PA; Wang, JY; Andrews, JC; Pianetta, P	TXM-Wizard: a program for advanced data collection and evaluation in full-field transmission X-ray microscopy	201	2012	281	287	10.1107/S0909049511049144
Leinweber, P; Kruse, J; Walley, FL; Gillespie, A; Eckhardt, KU; Blyth, RIR; Regier, T	Nitrogen K-edge XANES -: An overview of reference compounds used to identify 'unknown' organic nitrogen in environmental samples	193	2007	500	511	10.1107/S0909049507042513
Matz, W; Schell, N; Bernhard, G; Prokert, F; Reich, T; Claussner, J; Oehme, W; Schlenk, R; Dienel, S; Funke, H; Eichhorn, F; Betzl, M; Pröhl, D; Strauch, U; Hüttig, G; Krug, H; Neumann, W; Brendler, V; Reichel, P; Denecke, MA; Nitsche, H	ROBL -: a CRG beamline for radiochemistry and materials research at the ESRF	192	1999	1076	1085	10.1107/S0909049599010663
del Rio, MS; Canestrari, N; Jiang, F; Cerrina, F	SHADOW3: a new version of the synchrotron X-ray optics modelling package	191	2011	708	716	10.1107/S0909049511026306
Benfatto, M; Della Longa, S	Geometrical fitting of experimental XANES spectra by a full multiple-scattering procedure	189	2001	1087	1094	10.1107/S0909049501006422
Funke, H; Chukalina, M; Scheinost, AC	A new FEFF-based wavelet for EXAFS data analysis	186	2007	426	432	10.1107/S0909049507031901
Incardona, MF; Bourenkov, GP; Levik, K; Pieritz, RA; Popov, AN; Svensson, O	EDNA: a framework for plugin-based applications applied to X-ray experiment online data analysis	184	2009	872	879	10.1107/S0909049509036681
Gabadinho, J; Beteva, A; Guijarro, M; Rey-Bakaikoa, V; Spruce, D; Bowler, MW; Brockhauser, S; Flot, D; Gordon, EJ; Hall, DR; Lavault, B; McCarthy, AA; McCarthy, J; Mitchell, E; Monaco, S; Mueller-Dieckmann, C; Nurizzo, D; Ravelli, RBG; Thibault, X; Walsh, MA; Leonard, GA; McSweeney, SM	MxCuBE: a synchrotron beamline control environment customized for macromolecular crystallography experiments	183	2010	700	707	10.1107/S0909049510020005
Yamashita, H; Ichihashi, Y; Takeuchi, M; Kishiguchi, S; Anpo, M	Characterization of metal ion-implanted titanium oxide photocatalysts operating under visible light irradiation	183	1999	451	452	10.1107/S0909049598017257
Flot, D; Mairs, T; Giraud, T; Guijarro, M; Lesourd, M; Rey, V; van Brussel, D; Morawe, C; Borel, C; Hignette, O; Chavanne, J; Nurizzo, D; McSweeney, S; Mitchell, E	The ID23-2 structural biology microfocus beamline at the ESRF	179	2010	107	118	10.1107/S0909049509041168
Shin, JW; Eom, K; Moon, D	BL2D-SMC, the supramolecular crystallography beamline at the Pohang Light Source II, Korea	177	2016	369	373	10.1107/S1600577515021633

Potapkin, V; Chumakov, AI; Smirnov, GV; Celse, JP; Rüffer, R; McCammon, C; Dubrovinsky, L	The 57Fe Synchrotron Mossbauer Source at the ESRF	174	2012	559	569	10.1107/S0909049512015579
Dalba, G; Fornasini, P	EXAFS Debye-Waller factor and thermal vibrations of crystals	174	1997	243	255	10.1107/S0909049597006900
Prince, KC; Blyth, RR; Delaunay, R; Zitnik, M; Krempasky, J; Slezak, J; Camilloni, R; Avaldi, L; Coreno, M; Stefani, G; Furlani, C; de Simone, M; Stranges, S	The gas-phase photoemission beamline at Elettra	172	1998	565	568	10.1107/S090904959800065X
ELLIS, PJ; FREEMAN, HC	XFIT - AN INTERACTIVE EXAFS ANALYSIS PROGRAM	171	1995	190	195	10.1107/S0909049595006789
Knapp, M; Baehtz, C; Ehrenberg, H; Fuess, H	The synchrotron powder diffractometer at beamline B2 at HASYLAB/DESY: status and capabilities	163	2004	328	334	10.1107/S0909049504009367
Martínez-Criado, G; Villanova, J; Tucoulou, R; Salomon, D; Suuronen, JP; Labouré, S; Guilloud, C; Valls, V; Barrett, R; Gagliardini, E; Dabin, Y; Baker, R; Bohic, S; Cohen, C; Morse, J	ID16B: a hard X-ray nanoprobe beamline at the ESRF for nano-analysis	157	2016	344	352	10.1107/S1600577515019839
Zhou, ZY; Du, XW; Yang, JZ; Wang, YZ; Li, CY; Wei, S; Du, LL; Li, YY; Qi, F; Wang, QP	The vacuum ultraviolet beamline/endstations at NSRL dedicated to combustion research	155	2016	1035	1045	10.1107/S1600577516005816
Juanhuix, J; Gil-Ortiz, F; Cuní, G; Colldelram, C; Nicolás, J; Lidón, J; Boter, E; Ruget, C; Ferrer, S; Benach, J	Developments in optics and performance at BL13-XALOC, the macromolecular crystallography beamline at the Alba Synchrotron	155	2014	679	689	10.1107/S160057751400825X
Müller, O; Nachtegaal, M; Just, J; Lützenkirchen-Hecht, D; Frahm, R	Quick-EXAFS setup at the SuperXAS beamline for in situ X-ray absorption spectroscopy with 10 ms time resolution	154	2016	260	266	10.1107/S1600577515018007
Liermann, HP; Konôpková, Z; Morgenroth, W; Glazyrin, K; Bednarcik, J; McBride, EE; Petitgirard, S; Delitz, JT; Wendt, M; Bican, Y; Ehnes, A; Schwark, I; Rothkirch, A; Tischer, M; Heuer, J; Schulte-Schrepping, H; Kracht, T; Franz, H	The Extreme Conditions Beamline P02.2 and the Extreme Conditions Science Infrastructure at PETRA III	153	2015	908	924	10.1107/S1600577515005937
Borsboom, M; Bras, W; Cerjak, I; Detollenaere, D; van Loon, DG; Goedtkindt, P; Konijnenburg, M; Lassing, P; Levine, YK; Munneke, B; Oversluizen, M; van Tol, R; Vlieg, E	The Dutch-Belgian beamline at the ESRF	153	1998	518	520	10.1107/S0909049597013484
Piamonteze, C; Flechsig, U; Rusponi, S; Dreiser, J; Heidler, J; Schmidt, M; Wetter, R; Calvi, M; Schmidt, T; Pruchova, H; Krempasky, J; Quitmann, C; Brune, H; Nolting, F	X-Treme beamline at SLS: X-ray magnetic circular and linear dichroism at high field and low temperature	152	2012	661	674	10.1107/S0909049512027847
Benfatto, M; Della Longa, S; Natoli, CR	The MXAN procedure:: a new method for analysing the XANES spectra of metalloproteins to obtain structural quantitative information	150	2003	51	57	10.1107/S0909049502018137
Hesterberg, D; Zhou, WQ; Hutchison, KJ; Beauchemin, S; Sayers, DE	XAFS study of adsorbed and mineral forms of phosphate	148	1999	636	638	10.1107/S0909049599000370
Chollet, M; Alonso-Mori, R; Cammarata, M; Damiani, D; Defever, J; Delor, JT; Feng, YP; Glownia, JM; Langton, JB; Nelson, S; Ramsey, K; Robert, A; Sikorski, M; Song, S; Stefanescu, D; Srinivasan, V; Zhu, DL; Lemke, HT; Fritz, DM	The X-ray Pump-Probe instrument at the Linac Coherent Light Source	147	2015	503	507	10.1107/S1600577515005135
Lytle, FW	The EXAFS family tree: a personal history of the development of extended X-ray absorption fine structure	147	1999	123	134	10.1107/S0909049599001260
Winarski, RP; Holt, MV; Rose, V; Fuesz, P; Carbaugh, D; Benson, C; Shu, DM; Kline, D; Stephenson, GB; McNulty, I; Maser, J	A hard X-ray nanoprobe beamline for nanoscale microscopy	146	2012	1056	1060	10.1107/S0909049512036783
Liang, MN; Williams, GJ; Messerschmidt, M; Seibert, MM; Montanez, PA; Hayes, M; Milathianaki, D; Aquila, A; Hunter, MS; Koglin, JE; Schafer, DW; Guillet, S; Busse, A; Bergan, R; Olson, W; Fox, K; Stewart, N; Curtis, R; Miahnahri, AA; Boutet, S	The Coherent X-ray Imaging instrument at the Linac Coherent Light Source	145	2015	514	519	10.1107/S160057751500449X
Mathon, O; Beteva, A; Borrel, J; Bugnazet, D; Gatla, S; Hino, R; Kantor, I; Mairs, T; Munoz, M; Pasternak, S; Perrin, F; Pascarelli, S	The time-resolved and extreme conditions XAS (TEXAS) facility at the European Synchrotron Radiation Facility: the general-purpose EXAFS bending-magnet beamline BM23	144	2015	1548	1554	10.1107/S1600577515017786
Cianci, M; Bourenkov, G; Pompidor, G; Karpics, I; Kallio, J; Bento, I; Roessle, M; Cipriani, F; Fiedler, S; Schneider, TR	P13, the EMBL macromolecular crystallography beamline at the low-emittance PETRA III ring for high- and low-energy phasing with variable beam focusing	143	2017	323	332	10.1107/S1600577516016465
de Sanctis, D; Beteva, A; Caserotto, H; Dobias, F; Gabadinho, J; Giraud, T; Gobbo, A; Guijarro, M; Lentini, M; Lavault, B; Mairs, T; McSweeney, S; Petitdemange, S; Rey-Bakaikoa, V; Surr, J; Theveneau, P; Leonard, GA; Mueller-Dieckmann, C	ID29: a high-intensity highly automated ESRF beamline for macromolecular crystallography experiments exploiting anomalous scattering	143	2012	455	461	10.1107/S0909049512009715
Chen, LX; Rajh, T; Jäger, W; Nedeljkovic, J; Thurnauer, MC	X-ray absorption reveals surface structure of titanium dioxide nanoparticles	142	1999	445	447	10.1107/S090904959801591X
Vaughan, GBM; Wright, JP; Bytchkov, A; Rossat, M; Gleyzolle, H; Snigireva, I; Snigirev, A	X-ray transfocators: focusing devices based on compound refractive lenses	138	2011	125	133	10.1107/S0909049510044365
Kunz, M; MacDowell, AA; Caldwell, WA; Cambie, D; Celestre, RS; Domning, EE; Duarte, RM; Gleason, AE; Glossinger, JM; Kelez, N; Plate, DW; Yu, T; Zaug, JM; Padmore, HA; Jeanloz, R; Alivisatos, AP; Clark, SM	A beamline for high-pressure studies at the Advanced Light Source with a superconducting bending magnet as the source	138	2005	650	658	10.1107/S0909049505020959
Jiao, Y; Xu, G; Cui, XH; Duan, Z; Guo, YY; He, P; Ji, DH; Li, JY; Li, XY; Meng, C; Peng, YM; Tian, SK; Wang, JQ; Wang, N; Wei, YY; Xu, HS; Yan, F; Yu, CH; Zhao, YL; Qin, Q	The HEPS project	136	2018	1611	1618	10.1107/S1600577518012110
Parab, ND; Zhao, C; Cunningham, R; Escano, LI; Fezzaa, K; Everhart, W; Rollett, AD; Chen, LY; Sun, T	Ultrafast X-ray imaging of laser-metal additive manufacturing processes	136	2018	1467	1477	10.1107/S1600577518009554
Rosenbaum, G; Alkire, RW; Evans, G; Rotella, FJ; Lazarski, K; Zhang, RG; Ginell, SL; Duke, N; Naday, I; Lazarz, J; Molitsky, MJ; Keefe, L; Gonczy, J; Rock, L; Sanishvili, R; Walsh, MA; Westbrook, E; Joachimiak, A	The Structural Biology Center 19ID undulator beamline: facility specifications and protein crystallographic results	136	2006	30	45	10.1107/S0909049505036721
Shpyrko, OG	X-ray photon correlation spectroscopy	135	2014	1057	1064	10.1107/S1600577514018232
Gauthier, C; Solé, VA; Signorato, R; Goulon, J; Moguiline, E	The ESRF beamline ID26:: X-ray absorption on ultra dilute sample	135	1999	164	166	10.1107/S0909049598016835
Kieffer, J; Valls, V; Blanc, N; Hennig, C	New tools for calibrating diffraction setups	134	2020	558	566	10.1107/S1600577520000776
Strocov, VN; Wang, X; Shi, M; Kobayashi, M; Krempasky, J; Hess, C; Schmitt, T; Patthey, L	Soft-X-ray ARPES facility at the ADRESS beamline of the SLS: concepts, technical realisation and scientific applications	134	2014	32	44	10.1107/S1600577513019085
Nurizzo, D; Mairs, T; Guijarro, M; Rey, V; Meyer, J; Fajardo, P; Chavanne, J; Biasci, JC; McSweeney, S; Mitchell, E	The ID23-1 structural biology beamline at the ESRF	133	2006	227	238	10.1107/S0909049506004341
Paithankar, KS; Owen, RL; Garman, EF	Absorbed dose calculations for macromolecular crystals: improvements to RADDOSE	132	2009	152	162	10.1107/S0909049508040430
Proux, O; Nassif, V; Prat, A; Ulrich, O; Lahera, E; Biquard, X; Menthonnex, JJ; Hazemann, JL	Feedback system of a liquid-nitrogen-cooled double-crystal monochromator: design and performances	132	2006	59	68	10.1107/S0909049505037441
Tanaka, T; Kitamura, H	Analysis of figure-8-undulator radiation	132	1996	47	52	10.1107/S0909049596000155
Mezouar, M; Crichton, WA; Bauchau, S; Thurel, F; Witsch, H; Torrecillas, F; Blattmann, G; Marion, P; Dabin, Y; Chavanne, J; Hignette, O; Morawe, C; Borel, C	Development of a new state-of-the-art beamline optimized for monochromatic single-crystal and powder X-ray diffraction under extreme conditions at the ESRF	131	2005	659	664	10.1107/S0909049505023216
Morris, RJ; Zwart, PH; Cohen, S; Fernandez, FJ; Kakaris, M; Kirillova, O; Vonrhein, C; Perrakis, A; Lamzin, VS	Breaking good resolutions with ARP/wARP	131	2004	56	59	10.1107/S090904950302394X
Ren, Z; Bourgeois, D; Helliwell, JR; Moffat, K; Srajer, V; Stoddard, BL	Laue crystallography: coming of age	131	1999	891	917	10.1107/S0909049599006366
Beitlich, T; Kühnel, K; Schulze-Briese, C; Shoeman, RL; Schlichting, I	Cryoradiolytic reduction of crystalline heme proteins:: analysis by UV-Vis spectroscopy and X-ray crystallography	130	2007	11	23	10.1107/S0909049506049806
Nave, C; Hill, MA	Will reduced radiation damage occur with very small crystals?	130	2005	299	303	10.1107/S0909049505003274
Bouchard, R; Hupfeld, D; Lippmann, T; Neuefeind, J; Neumann, HB; Poulsen, HF; Rutt, U; Schmidt, T; Schneider, JR; Sussenbach, J; von Zimmermann, M	A triple-crystal diffractometer for high-energy synchrotron radiation at the HASYLAB high-field wiggler beamline BW5	130	1998	90	101	10.1107/S090904959701457X
Du, YH; Zhu, Y; Xi, SB; Yang, P; Moser, HO; Breese, MBH; Borgna, A	XAFCA: a new XAFS beamline for catalysis research	129	2015	839	843	10.1107/S1600577515002854
Rueff, JP; Ablett, JM; Céolin, D; Prieur, D; Moreno, T; Balédent, V; Lassalle-Kaiser, B; Rault, JE; Simon, M; Shukla, A	The GALAXIES beamline at the SOLEIL synchrotron: inelastic X-ray scattering and photoelectron spectroscopy in the hard X-ray range	128	2015	175	179	10.1107/S160057751402102X
Fischetti, R; Stepanov, S; Rosenbaum, G; Barrea, R; Black, E; Gore, D; Heurich, R; Kondrashkina, E; Kropf, AJ; Wang, S; Zhang, K; Irving, TC; Bunker, GB	The BioCAT undulator beamline 18ID: a facility for biological non-crystalline diffraction and X-ray absorption spectroscopy at the Advanced Photon Source	128	2004	399	405	10.1107/S0909049504016760
Mosselmans, JFW; Quinn, PD; Dent, AJ; Cavill, SA; Moreno, SD; Peach, A; Leicester, PJ; Keylock, SJ; Gregory, SR; Atkinson, KD; Rosell, JR	I18-the microfocus spectroscopy beamline at the Diamond Light Source	127	2009	818	824	10.1107/S0909049509032282
Nowell, H; Barnett, SA; Christensen, KE; Teat, SJ; Allan, DR	I19, the small-molecule single-crystal diffraction beamline at Diamond Light Source	125	2012	435	441	10.1107/S0909049512008801
Ingall, ED; Brandes, JA; Diaz, JM; de Jonge, MD; Paterson, D; McNulty, I; Elliott, WC; Northrup, P	Phosphorus K-edge XANES spectroscopy of mineral standards	125	2011	189	197	10.1107/S0909049510045322
Binsted, N; Hasnain, SS	State-of-the-art analysis of whole X-ray absorption spectra	124	1996	185	196	10.1107/S0909049596005651
Poulsen, HF; Garbe, S; Lorentzen, T; Jensen, DJ; Poulsen, FW; Andersen, NH; Frello, T; Feidenhansl, R; Graafsma, H	Applications of high-energy synchrotron radiation for structural studies of polycrystalline materials	123	1997	147	154	10.1107/S0909049597002021
Toraya, H; Hibino, H; Ohsumi, K	New powder diffractometer for synchrotron radiation with a multiple-detector system	123	1996	75	83	10.1107/S0909049595015500
Tavares, PF; Leemann, SC; Sjöström, M; Andersson, Å	The MAX IV storage ring project	122	2014	862	877	10.1107/S1600577514011503
Barla, A; Nicolás, J; Cocco, D; Valvidares, SM; Herrero-Martín, J; Gargiani, P; Moldes, J; Ruget, C; Pellegrin, E; Ferrer, S	Design and performance of BOREAS, the beamline for resonant X-ray absorption and scattering experiments at the ALBA synchrotron light source	120	2016	1507	1517	10.1107/S1600577516013461
Hettel, R	DLSR design and plans: an international overview	120	2014	843	855	10.1107/S1600577514011515
Beetz, T; Jacobsen, C	Soft X-ray radiation-damage studies in PMMA using a cryo-STXM	120	2003	280	283	10.1107/S0909049503003261
Winn, MD	An overview of the CCP4 project in protein crystallography: an example of a collaborative project	120	2003	23	25	10.1107/S0909049502017235
Ferreira, FF; Granado, E; Carvalho, W; Kycia, SW; Bruno, D; Droppa, R	X-ray powder diffraction beamline at D10B of LNLS:: application to the Ba2FeReO6 double perovskite	119	2006	46	53	10.1107/S0909049505039208
Yamashita, H; Harada, M; Misaka, J; Takeuchi, M; Ichihashi, Y; Goto, F; Ishida, M; Sasaki, T; Anpo, M	Application of ion beam techniques for preparation of metal ion-implanted TiO2 thin film photocatalyst available under visible light irradiation:: Metal ion-implantation and ionized cluster beam method	118	2001	569	571	10.1107/S090904950001712X
Gann, E; McNeill, CR; Tadich, A; Cowie, BCC; Thomsen, L	Quick AS NEXAFS Tool (QANT): a program for NEXAFS loading and analysis developed at the Australian Synchrotron	117	2016	374	380	10.1107/S1600577515018688
George, GN; Pickering, IJ; Pushie, MJ; Nienaber, K; Hackett, MJ; Ascone, I; Hedman, B; Hodgson, KO; Aitken, JB; Levina, A; Glover, C; Lay, PA	X-ray-induced photo-chemistry and X-ray absorption spectroscopy of biological samples	117	2012	875	886	10.1107/S090904951203943X
Teng, TY; Moffat, K	Primary radiation damage of protein crystals by an intense synchrotron X-ray beam	117	2000	313	317	10.1107/S0909049500008694
Bowler, MW; Nurizzo, D; Barrett, R; Beteva, A; Bodin, M; Caserotto, H; Delagenière, S; Dobias, F; Flot, D; Giraud, T; Guichard, N; Guijarro, M; Lentini, M; Leonard, GA; McSweeney, S; Oskarsson, M; Schmidt, W; Snigirev, A; von Stetten, D; Surr, J; Svensson, O; Pascal, TC; Mueller-Dieckmann, C	MASSIF-1: a beamline dedicated to the fully automatic characterization and data collection from crystals of biological macromolecules	116	2015	1540	1547	10.1107/S1600577515016604

Verbeni, R; Sette, F; Krisch, MH; Bergmann, U; Gorges, B; Halcoussis, C; Martel, K; Masciovecchio, C; Ribois, JF; Ruocco, G; Sinn, H	Semiconductor materials and radiation detection	116	2006	143	150	10.1107/S0909049505033339
	X-ray monochromator with 2x10(-8) energy resolution	116	1996	62	64	10.1107/S0909049595015883
Bech, M; Bunk, O; David, C; Ruth, R; Rifkin, J; Loewen, R; Feidenhans'l, R; Pfeiffer, F	Hard X-ray phase-contrast imaging with the Compact Light Source based on inverse Compton X-rays	115	2009	43	47	10.1107/S090904950803464X
Chen, S; Deng, J; Yuan, Y; Flachenecker, C; Mak, R; Hornberger, B; Jin, Q; Shu, D; Lai, B; Maser, J; Roehrig, C; Paunesku, T; Gleber, SC; Vine, DJ; Finney, L; VonOsinski, J; Bolbat, M; Spink, I; Chen, Z; Steele, J; Trapp, D; Irwin, J; Feser, M; Snyder, E; Brister, K; Jacobsen, C; Woloschak, G; Vogt, S	The Bionanoprobe: hard X-ray fluorescence nanoprobe with cryogenic capabilities	114	2014	66	75	10.1107/S1600577513029676
Seeck, OH; Deiter, C; Pflaum, K; Bertam, F; Beerlink, A; Franz, H; Horbach, J; Schulte-Schrepping, H; Murphy, BM; Greve, M; Magnussen, O	The high-resolution diffraction beamline P08 at PETRA III	113	2012	30	38	10.1107/S0909049511047236
Mathew, E; Mirza, A; Menhart, N	Liquid-chromatography-coupled SAXS for accurate sizing of aggregating proteins	112	2004	314	318	10.1107/S0909049504014086
Natoli, CR; Benfatto, M; Della Longa, S; Hatada, K	X-ray absorption spectroscopy: state-of-the-art analysis	112	2003	26	42	10.1107/S0909049502017247
Mokso, R; Schlepütz, CM; Theidel, G; Billich, H; Schmid, E; Celcer, T; Mikuljan, G; Sala, L; Marone, F; Schlumpf, N; Stampanoni, M	GigaFRoST: the gigabit fast readout system for tomography	111	2017	1250	1259	10.1107/S1600577517013522
Nave, C; Garman, EF	Towards an understanding of radiation damage in cryocooled macromolecular crystals	111	2005	257	260	10.1107/S0909049505007132
O'Neill, P; Stevens, DL; Garman, EF	Physical and chemical considerations of damage induced in protein crystals by synchrotron radiation: a radiation chemical perspective	111	2002	329	332	10.1107/S0909049502014553
Uruga, T; Tanida, H; Yoneda, Y; Takeshita, K; Emura, S; Takahashi, M; Harada, M; Nishihata, Y; Kubozono, Y; Tanaka, T; Yamamoto, T; Maeda, H; Kamishima, O; Takabayashi, Y; Nakata, Y; Kimura, H; Goto, S; Ishikawa, T	The XAFS beamline BL01B1 at SPring-8	111	1999	143	145	10.1107/S0909049598016173
Jeffries, CM; Graewert, MA; Svergun, DI; Blanchet, CE	Limiting radiation damage for high-brilliance biological solution scattering: practical experience at the EMBL P12 beamline PETRAIII	110	2015	273	279	10.1107/S1600577515000375
Giuliani, A; Jamme, F; Rouam, V; Wien, F; Giorgetta, JL; Lagarde, B; Chubar, O; Bac, S; Yao, I; Rey, S; Herbeaux, C; Marlats, JL; Zerbib, D; Polack, F; Réfrégiers, M	DISCO: a low-energy multipurpose beamline at synchrotron SOLEIL	110	2009	835	841	10.1107/S0909049509034049
Murray, J; Garman, E	Investigation of possible free-radical scavengers and metrics for radiation damage in protein cryocrystallography	110	2002	347	354	10.1107/S0909049502014632
NELMES, RJ; MCMAHON, MI	HIGH-PRESSURE POWDER DIFFRACTION ON SYNCHROTRON SOURCES	110	1994	69	73	10.1107/S0909049594006679
Yabashi, M; Tanaka, H; Ishikawa, T	Overview of the SACLA facility	109	2015	477	484	10.1107/S1600577515004658
Jiang, Z; Li, XF; Strzalka, J; Sprung, M; Sun, T; Sandy, AR; Narayanan, S; Lee, DR; Wang, J	The dedicated high-resolution grazing-incidence X-ray scattering beamline 8-ID-E at the Advanced Photon Source	109	2012	627	636	10.1107/S0909049512022017
Schnadt, J; Knudsen, J; Andersen, JN; Siegbahn, H; Pietzsch, A; Hennies, F; Johansson, N; Mårtensson, N; Öhrwall, G; Bahr, S; Mähl, S; Schaff, O	The new ambient-pressure X-ray photoelectron spectroscopy instrument at MAX-lab	108	2012	701	704	10.1107/S0909049512032700
Klysubun, W; Sombunchoo, P; Deenan, W; Kongmark, C	Performance and status of beamline BL8 at SLRI for X-ray absorption spectroscopy	107	2012	930	936	10.1107/S0909049512040381
Warwick, T; Ade, H; Kilcoyne, D; Kritscher, M; Tylisczcak, T; Fakra, S; Hitchcock, A; Hitchcock, P; Padmore, H	A new bend-magnet beamline for scanning transmission X-ray microscopy at the Advanced Light Source	107	2002	254	257	10.1107/S0909049502005502
Abernathy, DL; Grubel, G; Brauer, S; McNulty, I; Stephenson, GA; Mochrie, SGJ; Sandy, AR; Mulders, N; Sutton, M	Small-angle X-ray scattering using coherent undulator radiation at the ESRF	107	1998	37	47	10.1107/S0909049597015835
Thompson, SP; Parker, JE; Marchal, J; Potter, J; Birt, A; Yuan, F; Fearn, RD; Lennie, AR; Street, SR; Tang, CC	Fast X-ray powder diffraction on I11 at Diamond	106	2011	637	648	10.1107/S0909049511013641
Verbeni, R; Pylkkänen, T; Huotari, S; Simonelli, L; Vankó, G; Martel, K; Henriquet, C; Monaco, G	Multiple-element spectrometer for non-resonant inelastic X-ray spectroscopy of electronic excitations	106	2009	469	476	10.1107/S090904950901886X
Nass, K; Foucar, L; Barends, TRM; Hartmann, E; Botha, S; Shoeman, RL; Doak, RB; Alonso-Mori, R; Aquila, A; Bajt, S; Barty, A; Bean, R; Beyerlein, KR; Bublitz, M; Drachmann, N; Gregersen, J; Jönsson, HO; Kabsch, W; Kassemeyer, S; Koglin, JE; Krumrey, M; Mattle, D; Messerschmidt, M; Nissen, P; Reinhard, L; Sitsel, O; Sokaras, D; Williams, GJ; Hau-Riege, S; Timneanu, N; Caleman, C; Chapman, HN; Boutet, S; Schlichting, I	Indications of radiation damage in ferredoxin microcrystals using high-intensity X-FEL beams	104	2015	225	238	10.1107/S1600577515002349
Saitoh, Y; Fukuda, Y; Takeda, Y; Yamagami, H; Takahashi, S; Asano, Y; Hara, T; Shirasawa, K; Takeuchi, M; Tanaka, T; Kitamura, H	Performance upgrade in the JAEA actinide science beamline BL23SU at SPring-8 with a new twin-helical undulator	104	2012	388	393	10.1107/S0909049512006772
Dudin, P; Lacovig, P; Fava, C; Nicolini, E; Bianco, A; Cautero, G; Barinov, A	Angle-resolved photoemission spectroscopy and imaging with a submicrometre probe at the SPECTROMICROSCOPY-3.2L beamline of Elettra	104	2010	445	450	10.1107/S0909049510013993
Pascarelli, S; Mathon, O; Muñoz, M; Mairs, T; Susini, J	Energy-dispersive absorption spectroscopy for hard-X-ray micro-XAS applications	104	2006	351	358	10.1107/S0909049506026938
Heald, SM; Brewe, DL; Stern, EA; Kim, KH; Brown, FC; Jiang, DT; Crozier, ED; Gordon, RA	XAFS and micro-XAFS at the PNC-CAT beamlines	104	1999	347	349	10.1107/S090904959801677X
ELLEAUME, P	HELIOS - A NEW-TYPE OF LINEAR HELICAL UNDULATOR	104	1994	19	26	10.1107/S0909049594006370
Shen, Q; Bazarov, I; Thibault, P	Diffractive imaging of nonperiodic materials with future coherent X-ray sources	103	2004	432	438	10.1107/S0909049504016772
Wasserman, SR; Allen, PG; Shuh, DK; Bucher, JJ; Edelstein, NM	EXAFS and principal component analysis: a new shell game	103	1999	284	286	10.1107/S0909049599000965
Nagler, B; Arnold, B; Bouchard, G; Boyce, RF; Boyce, RM; Callen, A; Campell, M; Curiel, R; Galtier, E; Garofoli, J; Granados, E; Hastings, J; Hays, G; Heimann, P; Lee, RW; Milathianaki, D; Plummer, L; Schropp, A; Wallace, A; Welch, M; White, W; Xing, Z; Yin, J; Young, J; Zastrau, U; Lee, HJ	The Matter in Extreme Conditions instrument at the Linac Coherent Light Source	102	2015	520	525	10.1107/S1600577515004865
Yamamoto, S; Senba, Y; Tanaka, T; Ohashi, H; Hirono, T; Kimura, H; Fujisawa, M; Miyawaki, J; Harasawa, A; Seike, T; Takahashi, S; Nariyama, N; Matsushita, T; Takeuchi, M; Ohata, T; Furukawa, Y; Takeshita, K; Goto, S; Harada, Y; Shin, S; Kitamura, H; Kakizaki, A; Oshima, M; Matsuda, I	New soft X-ray beamline BL07LSU at SPring-8	101	2014	352	365	10.1107/S1600577513034796
Frenkel, AI	Solving the structure of nanoparticles by multiple-scattering EXAFS analysis	101	1999	293	295	10.1107/S0909049598017786
McDonald, SA; Marone, F; Hintermüller, C; Mikuljan, G; David, C; Pfeiffer, F; Stampanoni, M	Advanced phase-contrast imaging using a grating interferometer	100	2009	562	572	10.1107/S0909049509017920
Polikarpov, I; Perles, LA; de Oliveira, RT; Oliva, G; Castellano, EE; Garratt, RC; Craievich, A	Set-up and experimental parameters of the protein crystallography beamline at the Brazilian National Synchrotron Laboratory	100	1998	72	76	10.1107/S0909049597014684
